# Nonlinear Stabilization Controller for the Boost Converter with a Constant Power Load in Both Continuous and Discontinuous Conduction Modes

**DOI:** 10.3390/mi12050522

**Published:** 2021-05-06

**Authors:** Juan Gerardo Parada Salado, Carlos Alonso Herrera Ramírez, Allan Giovanni Soriano Sánchez, Martín Antonio Rodríguez Licea

**Affiliations:** 1 Department of Electronics, Celaya Institute of Technology, Celaya 38010, Mexico; d1903018@itcelaya.edu.mx; 2 Robotic Engineering Department, Polytechnic University of Guanajuato, Cortazar 38496, Mexico; aherrera@upgto.edu.mx; 3 CONACYT-Celaya Institute of Technology, Celaya 38010, Mexico; allan.soriano@itcelaya.edu.mx

**Keywords:** constant power load, boost converter, discontinuous conduction mode, nonlinear control, switched system

## Abstract

The operation of Boost converters in discontinuous conduction mode (DCM) is suitable for many applications due to the, among other advantages, inductor volume reduction, high efficiency, paralleling, and low cost. Uses in biomedicine, nano/microelectromechanical, and higher power systems, where wide ranges of input/output voltage and a constant power load (CPL) can coexist, are well-known examples. Under extremely wide operating ranges, it is not difficult to change to a continuous conduction mode (CCM) operation, and instability, chaos, or bifurcations phenomena can occur regardless of the conduction mode. Unfortunately, existing control strategies consider a single conduction mode or linearized models because only slight resistive/CPL power level or input/output voltage variations (and no conduction mode changes) were expected. In this paper, new mathematical models for the Boost converter (with resistive or CPL) that are conduction mode independent are presented and validated. Since the open-loop dynamics of the proposed CPL model is unstable, a nonlinear control law capable of stabilizing the boost converter regardless of the conduction mode is proposed. A stability analysis based on a common-Lyapunov function is provided, and numerical and experimental tests are presented to show the proposal’s effectiveness.

## 1. Introduction

It is well-known that the Boost converter can operate in three modes, named CCM, critical conduction mode (CRM), and DCM, related to the energy stored in the inductor. In CCM/DCM, the stored energy is strictly/non strictly greater than zero, and CRM stands for zero energy during an infinitesimal period. Many authors have considered the Boost converter’s modeling and stabilization in CCM with a resistive load since no wide variations or constant power phenomena were expected at the load side. Some only mention that DCM operation should be avoided or treated with special care, and others have limited the DCM operation’s study to its stationary behavior [1,2,3,4,5,6]. A brief survey of relevant DCM, CRM, and CCM models and controllers for the Boost converter is presented in the following.

The authors in [7] developed a low-output voltage Boost converter operating in DCM and designed a controller to stabilize the output by a steady-state analysis. Some authors analyzed the power quality in DCM operation for converters and rectifiers, for instance, in [8,9,10,11,12,13]. However, they based their analysis on steady-state behavior. Small signal linear models for DCM operation of the Boost converter, including power losses, were developed in [14]; however, the obtained transfer function includes many parameters that are not easy to acquire. In [15], a frequency control to operate the Boost converter in CRM was proposed. The authors based their study on a linear model of the Boost converter operating in DCM, obtained by a small-signal analysis. On the other hand, in [16], a numerical PSPICE implementation of an isolated full-bridge converter reproduced some CCM and DCM phenomena.

In [17], a nonlinear controller for DCM operation of the Boost converter was developed, whereby the authors obtained such controller from the discrete-time difference-approximation equations and the steady-state solution of the system. The authors in [18] proposed a controller for the output current of the Boost converter operating in DCM, using a steady-state estimation of the dynamics.

In [19], the authors developed a conduction mode independent (CMI) model for the non-inverting Buck-Boost converter. However, the proposed model depends on the inductor’s discharge period, which is an implicit function of the duty cycle and parameters and cannot be extended to the Boost converter. Regardless of the previous, a back-stepping controller for the Buck-Boost converter with a resistive load (non-CPL) was developed.

Some authors have stated unwanted phenomena during the transition between conduction modes. In [20,21], proofs of open-loop bifurcation phenomena in the DCM-CCM boundary were provided. Chaos and bifurcation behaviors for the boost converter were also demonstrated in [22]. Controllers for other converters operating in DCM or CCM with no CPL were developed in [23,24,25].

Furthermore, it has been reported that a CPL can destabilize the dynamics of the Boost converter in any conduction mode. Recently developed control strategies considered a CPL with single conduction modes or linearized models [26,27,28,29,30,31,32,33]. Unfortunately, it is not difficult to achieve DCM to CCM changes in a Boost converter, with both a resistive load or a CPL and in addition, with a CPL, the conduction mode depends on their power level and output voltage/current. Moreover, linearized models are accurate only in a small region containing the operating point.

It is worth mentioning the work in [34], the authors reported that the Boost converter operating in DCM with a CPL is stable during steady-state. However, the transient stage and CCM to CCM changes cannot be neglected for applications in which wide ranges of input/output voltage and a constant power load (CPL) can coexist.

The authors in [35] presented a current control for the Boost converter feeding a CPL and included a passive compensation (paralleled RC network). They based their analysis on small-signal models valid only in a small region containing the operating point.

From the above state of the art, one can find that the complicated scenario that includes CPLs, and conduction mode changes in the Boost converter, has not been formally studied. The models presented until now are small-signal or steady-state. Table 1 summarizes relevant proposals for converters operating with a CPL or different conduction modes; the checkmark symbol means that the research considers the characteristic. There is a gap for the Boost converter’s modeling and control with a CPL, operating in both DCM and CCM. Unfortunately, in biomedicine, microelectromechanical, and other applications, wide CPL and input/output voltage variation ranges could induce changes in the conduction mode and stability properties.

Hence, the contributions of this paper are as follows. Precise Boost converter CMI models (CPL-switched, CPL non-switched, resistive load switched, and resistive load non-switched) are presented. Through such models, it is shown that the Boost converter’s operating point with a CPL is potentially unstable in open-loop; this has not been demonstrated from a CMI perspective to the authors’ knowledge. Therefore, a nonlinear stabilizing controller is developed for the CMI model with a CPL. A stability analysis based on a common-Lyapunov function is provided, and numerical and experimental tests are presented to show the proposal’s effectiveness. The controller does not depend on frequency control but the CPL power level and the output current, making it easy to implement.

This document is organized as follows. Section 2 presents the development of the mathematical models, and Section 3 its validation. Section 4 is devoted to instability demonstration for the Boost converter with a CPL. In Section 5, the controller design is presented, and in Section 6, numerical and experimental tests of the closed-loop system are presented. Finally, some conclusions are presented in Section 7.

## 2. CMI Model of the Boost Converter

**Figure 1:**
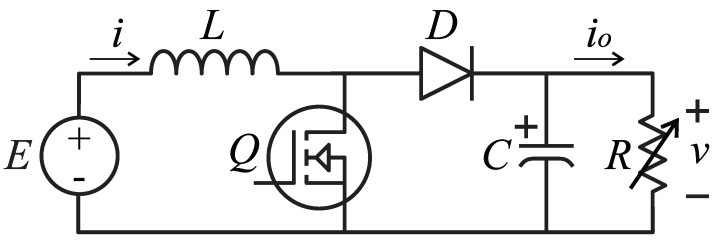
Basic Boost converter schematic with a variable resistive load whose value is modified to achieve a constant output power (CPL).

Consider the Boost converter schematic of Figure 1. Let us begin by introducing the well-known simplified mathematical model of the Boost converter with a CPL, obtained by an averaging technique in CCM, with infinite switching frequency as well as ideal components [1]:

(1) $$L\frac{di}{dt}=-\left(1-u_{1}\right)v+E$$

(2) $$C\frac{dv}{dt}=\left(1-u_{1}\right)i-\frac{P}{v}$$

where *L*, *C*, and *P* are the inductance, capacitance, and CPL power demand, respectively; *v* is the averaged output voltage, *i* is the averaged current flowing through the inductor, $R=v^{2}/P$ where *P* is the output power, and $u_{1}$ is the duty cycle of the PWM signal in *Q*. Note that v=0 implies an indeterminate form in Equation (2), characteristic for the Boost converter with a CPL. Since protective circuits can avoid a high output current, it is reasonable to consider the output voltage and current, both greater than zero, for analysis purposes.

On the other hand, using $R=v^{2}/P$ in the well-known conduction-mode-inequality for the Boost converter [36], DCM occurs if:

(3) $$k_{crit}\left(v\right)=\frac{2LfP}{v^{2}}<u_{1}{\left(1-u_{1}\right)}^{2}$$

where *f* is the operating frequency of the PWM. In principle, the Boost converter cannot be readily designed to operate permanently in CCM or DCM with a CPL. If *P* varies to smaller values (low load), the converter could operate in DCM; conversely, a DCM to CCM change can occur if the CPL level has a high value. Recall that at this point, *v* and *i* both represent averaged values.

**Figure 2:**
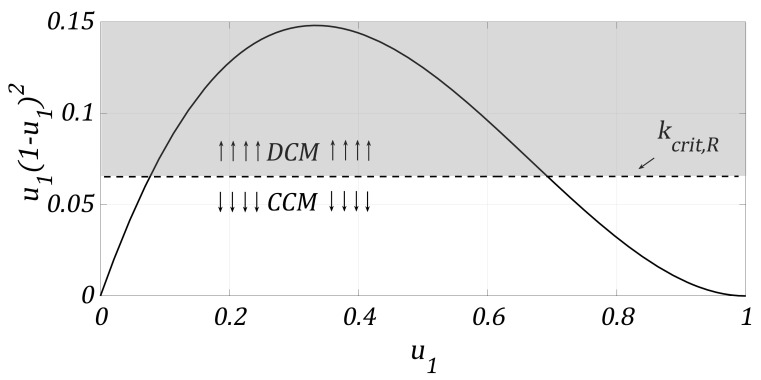
DCM/CCM dependence on *u* and $k_{crit,R}$.

In an ideal resistive load scenario, a designer tries to ensure that the range for $u_{1}$ is as wide as possible to ensure either DCM or CCM (selecting the components to achieve $k_{crit,R}=2fL/R<u{\left(u-1\right)}^{2}$ or $k_{crit,R}=2fL/R>u{\left(u-1\right)}^{2}$, respectively). Figure 2 illustrates the conduction modes as a function of $u_{1}$ and $k_{crit,R}$. It is easy to notice that even in such a scenario, it is impossible to ensure a single conduction mode if the load is not a variable to be altered/bounded at will. Furthermore, the components’ degradation or aging can alter $k_{crit,R}$, inducing a conduction mode change; hence, the dynamical model of Equations (1) and (2) obtained for CCM operation would not be appropriate for designing a controller. Indeed, many authors have demonstrated the bifurcation phenomena during the CCM to DCM change [20,21,22].

Therefore, to stabilize/regulate the Boost converter with a CPL in any conduction mode, description (1) and (2) is not adequate. In the following, a complementary averaged model for DCM is used to obtain later CMI models for the Boost converter with/without a CPL. As far as the authors know, there are no CMI models such as the one described below.

**Figure 3:**
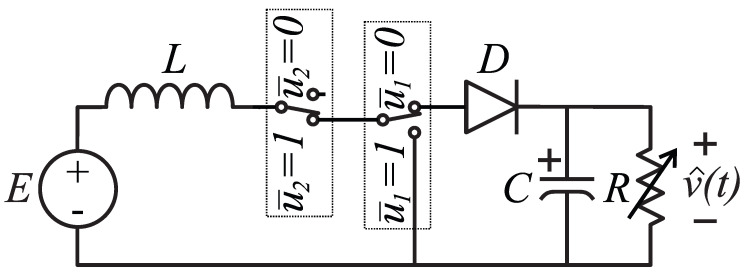
Idealization of the Boost converter operating in DCM through two ideal switches.

**Figure 4:**
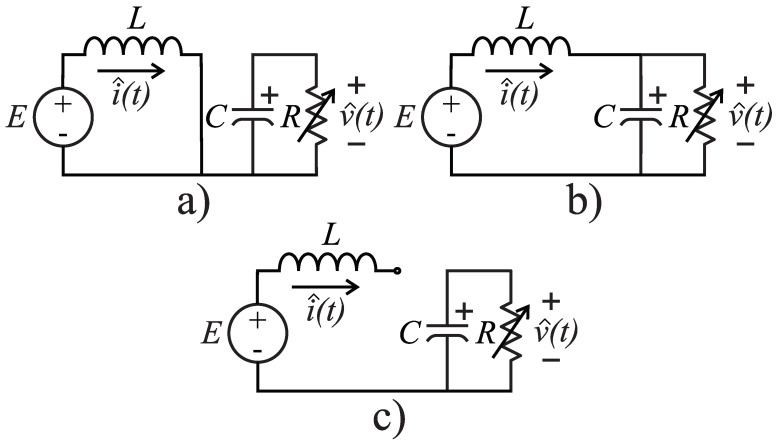
Illustration of the inductor current flow modes: (**a**) Charging, (**b**) discharging, and (**c**) holding.

For a DCM operation of the Boost converter, one can idealize *Q* as two switches ${\bar{u}}_{1}$, ${\bar{u}}_{2}$ as described in Figure 3; ${\bar{u}}_{2}=0$ stands for the zero current through the inductor (*L*). Reproducing the averaging methodology in [1] to obtain a DCM model, the three operating modes depicted in Figure 4 are possible. Mathematical expressions for each operating mode are presented in Table 2; note that *X* means “do not care” because ${\bar{u}}_{2}=0$ nullifies the current flow through *L*, and hence ${\bar{u}}_{1}$ has no effect on the equations. Note also that $\hat{v}$ and $\hat{i}$ are used to differentiate them from the averaged values *v* and *i*.

**Figure 5:**
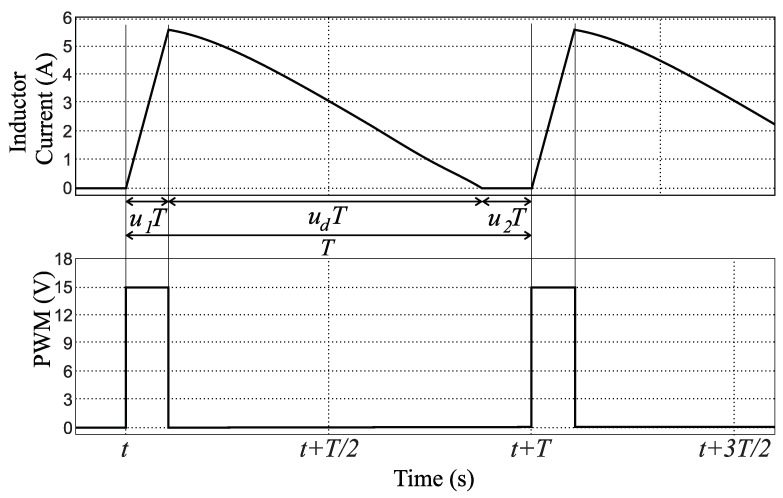
Exemplification of the inductor current behavior in DCM concerning the triggering on *Q*. PWM stands for the voltage signal in the gate pin.

An elementary analysis allows obtaining an averaged model of the charging, discharging, and holding operating modes as:

(4) $$L\frac{di}{dt}=-\left(1-u_{1}\right)v+E\left(1+2u_{1}u_{2}\right)$$

(5) $$C\frac{dv}{dt}=\left(1-u_{1}\right)i-\frac{P}{v}$$

where $u_{1}\in \left[0,1\right]$ is the duty cycle (percentage of *T* in charging mode with T=1/f as the PWM period), and $u_{2}$ is the percentage of *T* in holding mode (zero inductor current); these periods should not be confused with ${\bar{u}}_{1}$, ${\bar{u}}_{2}$ since these last only represent the switches in Figure 3. See Table 2 and Figure 5 for an illustration; $u_{d}$ is used to describe the period’s percentage in discharge mode. Note that $u_{2}=0$ for CCM and $u_{2}>0$ for DCM; hence, there is no ambiguity on using $u_{1}$, *v*, and *i* from the CCM model of Equations (1) and (2), for this model also.

From inductor and diode current waveforms in steady-state, it is well known that the load current is [36]:

(6) $$\frac{v}{R}=\frac{P}{v}=\frac{Eu_{1}u_{d}}{2fL}.$$

Furthermore, a time balance over the inductor current can be represented as (see Figure 5):

(7) $$T=u_{1}T+u_{d}T+u_{2}T$$

such that:

(8) $$u_{2}=1-u_{1}-u_{d}=1-u_{1}-\frac{2PLf}{Evu_{1}}.$$

Then, it is easy to see that:

(9) $$u_{2}=1-u_{1}-\frac{2PLf}{Evu_{1}}$$

is a $C^{1}$-diffeomorphism (for the inverse, it is enough to take only the positive root to have a bijection; this inverse is differentiable) for Equations (4) and (5) with v>0 and $1\geq u_{1}>0$ ($u_{1}=0$ is a trivial case), allowing to eliminate the dependency on $u_{2}$ [37]:

(10) $$L\frac{di}{dt}=-\left(1-u_{1}\right)v-\frac{4PLf}{v}+\left(1+2u_{1}-2u_{1}^{2}\right)E$$

(11) $$C\frac{dv}{dt}=\left(1-u_{1}\right)i-\frac{P}{v}.$$

The following section provides validations showing that the result of this transformation is quantitatively favorable in terms of the mean squared error (MSE).

By comparison of Equations (10) and (11) with Equations (1) and (2) and from inequality (3), one can design/approximate (recall that the CCM and DCM models were obtained using an infinite frequency consideration, while the change between conduction modes (3) is obtained from a steady-state analysis. Since a common Lyapunov function is used to design a controller and demonstrate stability even with arbitrary switching, it is not relevant to obtain a precise design of the switching signal; these following switching signals are presented for completeness purposes) a switching signal as $\gamma \left(u_{1},v\right)\in \left\{0,1\right\}$:

(12) $$\gamma \left(u_{1},v\right)≜0.5\left(1+sign\left(\left(u_{1}{\left(1-u_{1}\right)}^{2}-\frac{2LfP}{v^{2}}\right)\right)\right)$$

where:

(13) $$sign\left(x\right)=\left\{\begin{array}{cc} -1, & x<0\\ 0, & x=0\\ 1, & x>0 \end{array}\right..$$

That is, $\gamma (u_{1},v)=1$ for DCM, and $\gamma (u_{1},v)=0$ for CCM; the switched model is then:

(14) $$L\frac{di}{dt}=-\left(1-u_{1}\right)v-\frac{4PLf}{v}\gamma +\left(1+2\gamma u_{1}-2\gamma u_{1}^{2}\right)E$$

(15) $$C\frac{dv}{dt}=\left(1-u_{1}\right)i-\frac{P}{v}.$$

Alternatively, one can approximate the switching signal with a $C^{\infty }$ function 0≤ρ≤1 to obtain a continuous (non-switched) CMI model of the Boost converter:

(16) $$\rho \left(u_{1},v\right)=0.5\left(1+tanh\left(a\left(u_{1}{\left(1-u_{1}\right)}^{2}-\frac{2LfP}{v^{2}}\right)\right)\right)$$

with a≫1, and the switched model is:

(17) $$L\frac{di}{dt}=-\left(1-u_{1}\right)v-\frac{4PLf}{v}\rho +\left(1+2\rho u_{1}-2\rho u_{1}^{2}\right)E$$

(18) $$C\frac{dv}{dt}=\left(1-u_{1}\right)i-\frac{P}{v}.$$

Note that for γ=0 and ρ=0, the operating point congruently coincides with that of the classic CCM model.

For the non-CPL case (resistive load), the switched/CMI model can be obtained by a similar procedure:

(19) $$L\frac{di}{dt}=-\left(1-u_{1}+\frac{4Lf}{R}\sigma \right)v+\left(1+2\sigma u_{1}-2\sigma u_{1}^{2}\right)E$$

(20) $$C\frac{dv}{dt}=\left(1-u_{1}\right)i-\frac{v}{R}$$

where:

(21) $$\sigma \left(u_{1}\right)≜0.5\left(1+sign\left(\left(u_{1}{\left(1-u_{1}\right)}^{2}-\frac{2Lf}{R}\right)\right)\right),$$

or alternatively:

(22) $$L\frac{di}{dt}=-\left(1-u_{1}+\frac{4Lf}{R}\varphi \right)v+\left(1+2\varphi u_{1}-2\varphi u_{1}^{2}\right)E$$

(23) $$C\frac{dv}{dt}=\left(1-u_{1}\right)i-\frac{v}{R}$$

with,

(24) $$\varphi \left(u_{1}\right)=0.5\left(1+tanh\left(a\left(u_{1}{\left(1-u_{1}\right)}^{2}-\frac{2Lf}{R}\right)\right)\right).$$

## 3. Validation of the CMI Model for the Boost Converter

In this section, the validations for the non-CPL model developed in Equations (19) and (20) are presented (validation of the model of Equations (22) and (23) is not presented here because clearly, it provides a very smooth CCM-DCM switching in comparison with the model of Equations (19) and (20); the dynamic behavior is almost the same for other conditions except during this switching); this is done by comparing the classic CCM model in [1] (Equations (1) and (2) in this paper), and the s-domain linear DCM model presented in [14]. PSIM 2020a is used to generate the control group data.

**Figure 6:**
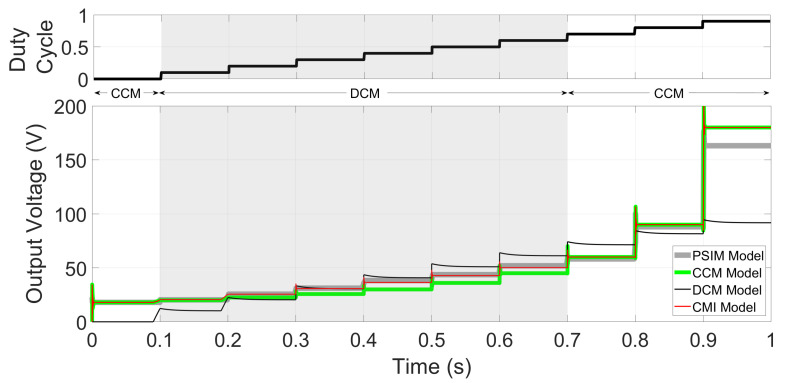
Comparative of the output voltage for PSIM (averaged), CCM, DCM, and CMI models, using steps from $u_{1}=0.0$ to $u_{1}=0.90$.

**Figure 7:**
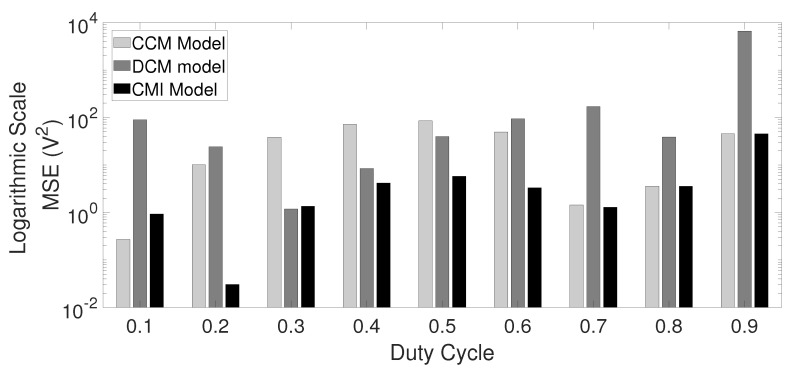
Voltage mean-squared error for CCM, DCM, and CMI models, in logarithmic scale. The CMI model presented in this paper provides the lowest MSE in almost all the $u_{1}$ range.

Consider E=100 V, L=15 μH, C=100 μF, R=10 Ω, and f=20 kHz; $0.0693<u_{1}<0.7091$ values allow DCM while other values allow CCM operation. Zero initial conditions and steps from $u_{1}=0.10$ to $u_{1}=0.90$ are introduced. It can be easily noted from Figure 6 that the CMI model shows minimum voltage error compared to the other models. The DCM model shows minimal error only around the linearization point ($u_{1}=0.35$, which is in the middle of the DCM range). The CCM classic model is inaccurate if the converter operates in DCM (shadowed area in Figure 2). To show the error quantitatively, Figure 7 shows a comparative plot of the MSE against $u_{1}$ for the three models. MSE for the inductor current, although not presented here, shows very similar results.

**Figure 8:**
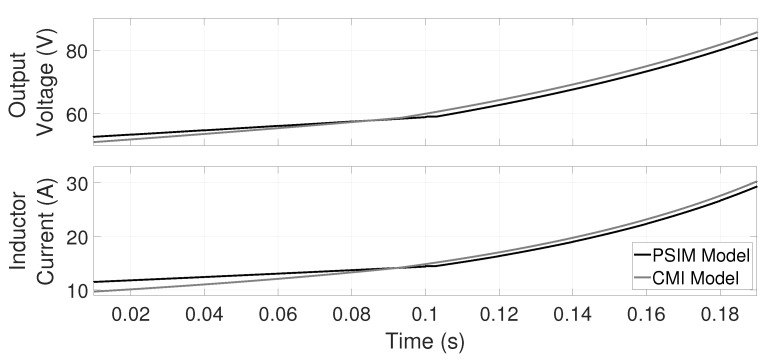
A comparison of the output voltages and inductor’s currents obtained with the PSIM (averaged) and CMI models for a ramp input with unitary slope. The model presented in this paper can accurately follow the response-curves change during the DCM to CCM switch.

Figure 8 shows the comparatives of *v* and *i* during the switch/change between conduction modes as a function of $u_{1}$. A slow ramp with a positive slope is introduced to force the DCM to CCM change about 0.1 s, showing no impulsive effects in both PSIM and CMI model dynamics. Note that the model presented in this paper can accurately follow the slope change during the DCM to CCM switch; this is not the case for the classic CCM model or the DCM model.

**Figure 9:**
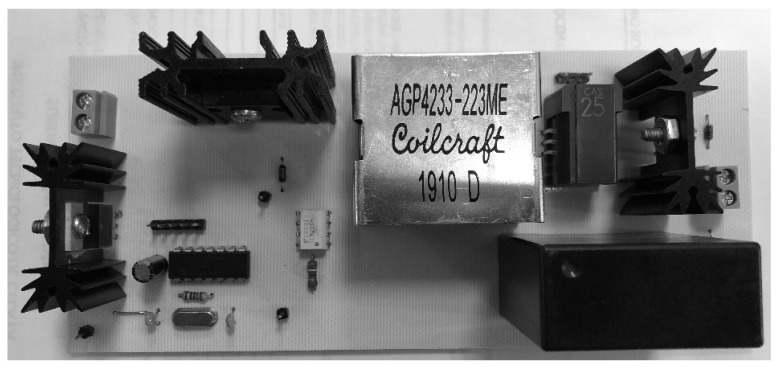
PCB used for the experimental tests presented in this paper.

**Figure 10:**
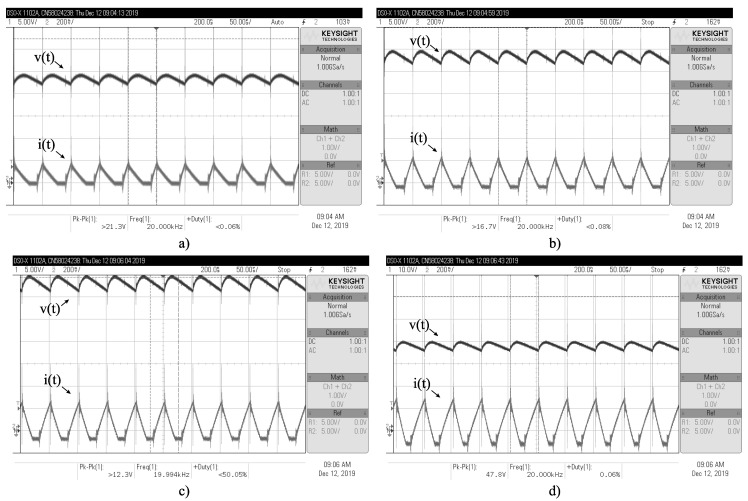
Output voltage and inductor current for the experimental validation, with (**a**) u=0.1, (**b**) u=0.2, (**c**) u=0.3, and (**d**) u=0.4. The current error is at an average of 0.223 *A*, and the RMS voltage error is approximately 3.12 *V*.

As an additional validation, an experimental 200-W Boost converter is built with the parameters mentioned above (see Figure 9) to validate the non-CPL CMI model. The inductor’s current is measured with a current transducer from LEM USA Inc., and the load (resistive for this test) is emulated with a BK PRECISION-8510 electronic load. Figure 10 shows representative output voltages and inductor currents for several values of $u_{1}$; from experimental and CMI model simulation data, the current error is on average 0.223 A, and the RMS voltage error is approximately 3.12 V for $u_{1}=0.1$ up to $u_{1}=0.8$.

**Figure 11:**
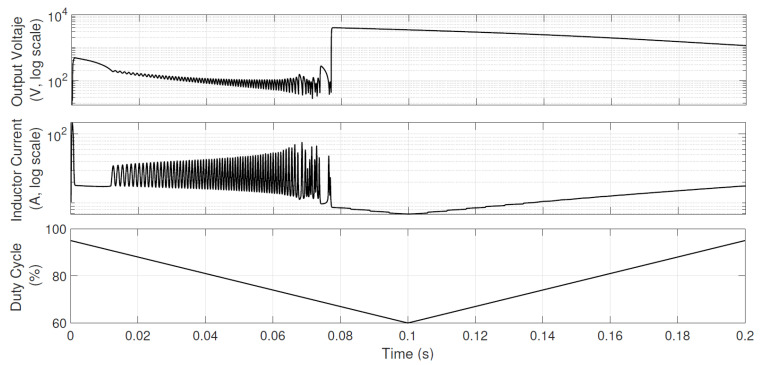
Illustration of open-loop, unstable behavior dependence on $u_{1}$. For this example, the same previous parameters are used with P=500 W. Average output voltage and inductor current are obtained by PSIM (averaged values) and shown in logarithmic scales. $u_{1}$ is introduced as a (slow-dynamics) triangular wave to show how values lower than approximately 72% destabilizes the converter with a CPL; even worse, even if the active cycle increases again, stability cannot be recovered. Note that for high values of $u_{1}$, the voltage and current do not show large amplitude oscillations, but as the cycle decreases, the oscillations increase considerably.

It is important to mention at this point that the CMI model with a CPL can hardly be validated experimentally in an open-loop scenario (essentially, for high power and low duty-cycle values, the converter’s operating point becomes unstable, as illustrated in Figure 11). As shown below, a CPL induces instability, and it is preferable and less risky to validate a stable dynamic instead.

## 4. Open-Loop Instability of the Boost Converter Dynamics with a CPL

Consider the dynamic system represented by Equations (14) and (15). The following shows that an operating point of such a system can be unstable either in DCM or CCM operation.

**Proposition** **1.** *At least, one operating point of the dynamic system represented by the Equations (14) and (15), is Lyapunov-unstable for P,L>0.*

**Proof.**  See Appendix A.1. □

It is worth mentioning that different operating points may be open-loop stable; however, in general, open-loop stability cannot be ensured by a constant control input. The interested reader will surely obtain parameters and constant values of $u_{1}$ for which the operating point is stable. However, the question remains whether there is a control signal $u_{1}$ capable of avoiding unstable system dynamics.

## 5. Nonlinear Stabilization Controller for the Boost Converter with a CPL for CMI Operation

This section shows that a nonlinear control law can stabilize the system represented by Equations (14) and (15). At present, stability for general nonlinear switched systems under arbitrary switching can be ensured only by a common Lyapunov function approach; see, for instance [38,39] and the references therein.

**Theorem** **1.** *Let P>0, v>0, i>0, and v≥E. The gain scheduling control law:*

(25) $$1-u_{1}=\frac{k_{1}P}{v}-k_{2}P-\frac{k_{3}\dot{v}}{v^{2}}-k_{4}\dot{v},$$

(26) $$\begin{array}{c} \;using\;for\;\gamma =1\left\{\begin{array}{c} k_{1}=\frac{3k_{3}-12CELf}{8CPLf}\\ k_{2}>0\\ k_{3}>0\;(small\;enough)\\ k_{4}=\frac{3k_{3}}{8LPf} \end{array}\right., \end{array}$$

(27) $$\begin{array}{c} \;and\;for\;\gamma =0\left\{\begin{array}{c} k_{1}>0\;(small\;enough)\\ k_{2}>\frac{1}{P}\\ k_{3}=0\\ k_{4}=0 \end{array}\right.,\; \end{array}$$

*stabilizes the switched system* (14) *and* (15) *for an arbitrary switching law.*

**Proof.**  See the Appendix A.2. □

It is worth mentioning that the small enough constants can be selected by establishing an upper bound for *i*; this is feasible because the inductor has a saturation current.

## 6. Closed-Loop Numerical and Experimental Validations

**Figure 12:**
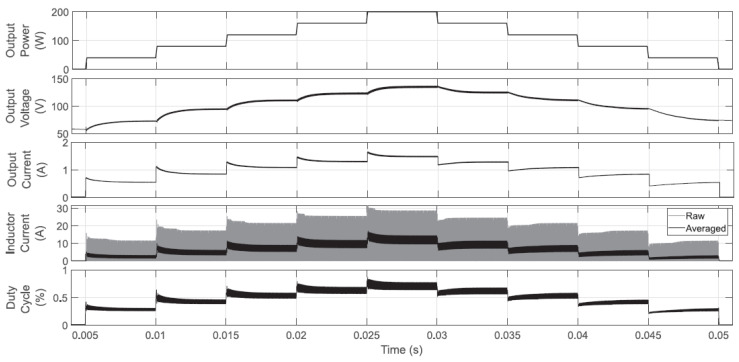
CPL sweep in closed-loop operation. The duty cycle never reaches the upper limit (set to 0.95), and a DCM operation is achieved at almost all times.

Firstly, numerical validations for the system operating in a closed-loop with the proposed controller are presented; this is done by using two CPL sweeps and the parameters established in Section 3. The first sweep consists of introducing 40 W steps in the CPL level, from 0 up to 200 W (PSIM), as shown in the upper plot of Figure 12. The proposed controller in Equation (25) is used to calculate the Boost converter input control $u_{1}$, and the dynamic behavior is shown in the lower plots of such a figure. The second and third plots show the output voltage and output current dynamics, respectively; the controller adequately adapts voltage and current levels to achieve the CPL demand. Since power levels are achievable, the controller, besides stabilizing the converter, provides stable operating points for voltage and currents (inductor and output). In this test, the boost converter operates almost in DCM at all times because the duty cycle is calculated within zero and 0.85. For completeness purposes in the two last plots of Figure 12, the inductor current and the calculated duty cycle are shown. It is worth highlighting that using gains that do not accomplish conditions (26) and (27) or using regular proportional-integral controllers, unstable voltage, and current levels are obtained (not shown here). Regularly in such situations, the voltage suddenly increases, and the current falls, and the probability of devices’ damage is very high.

**Figure 13:**
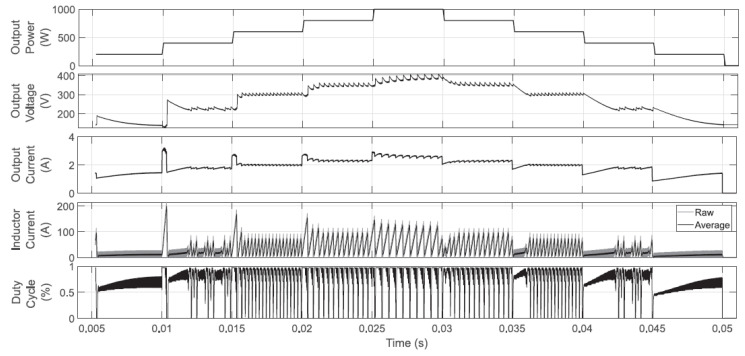
CPL sweep in the closed-loop operation of the Boost converter. The duty cycle reaches the upper limit (set to 0.95), and it is not possible to compensate for the small oscillations in output voltage and current. This is an inherent characteristic of the Boost converter.

A second CPL sweep of larger amplitudes is introduced to corroborate the controller’s operation during the conduction mode changes. The associated dynamic behavior is presented in Figure 13. Note that in this case, up to 1 kW is demanded, and inductor-current peaks up to 200 A are reached. There are clear commutations from DCM to CCM and vice versa, in addition to significant changes in the inductor current. Despite the above, the CPL can be powered with acceptable voltage and current variations at the output. Although in the previous sweep, it was confirmed that fast changes to CCM were obtained, this test clearly proves that CCM is reached for long periods. In this severe scenario, the voltage gain limit of the converter is intentionally reached; oscillations are expected because the controller looks to increase the current to feed extremely high CPL levels.

**Figure 14:**
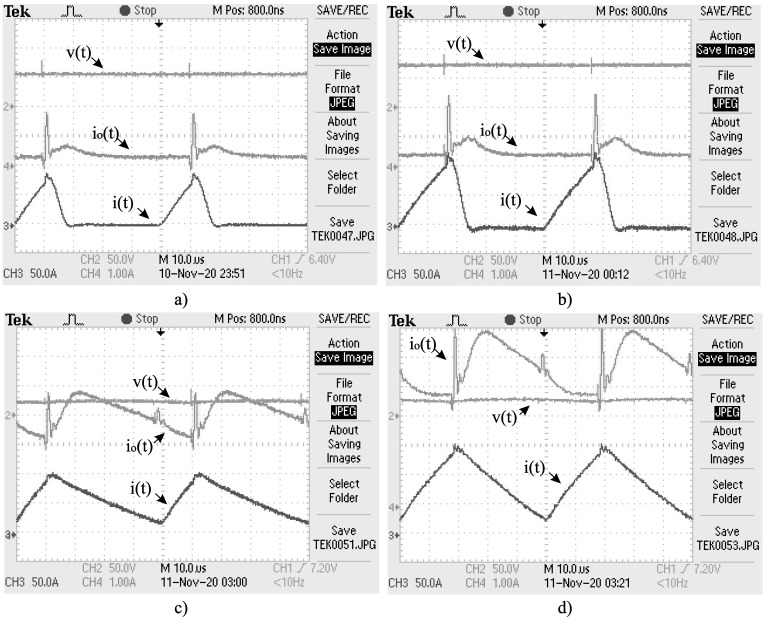
Output voltage (*v*), output current (io), and inductor current (*i*) for the Boost converter operating in closed-loop, with a CPL power demand of (**a**) 20, (**b**) 40, (**c**) 100, and (**d**) 135 W. 50 V/div is used for *v*, 1 A/div for io, and 5 A/div for *i* (the probe is configured for x/10). The output voltage and both currents are stabilized in the four scenarios despite the conduction mode type.

In the following, the results of the experimental tests in closed-loop are presented. The same Boost converter used for model validation in Section 3 is employed to perform the following experiments; however, the measurement of the output voltage and current must be performed to calculate the controller action (Equation (25)). The BK PRECISION-8510 electronic load is now programmed to consume constant power levels of 25, 50, 100, and 150 W. Figure 14 shows the four scenarios described; note that these signals are not averaged and are shown in different scales. At all the levels of CPL’s power demand, it is possible to stabilize both the output voltage and the inductor current, and the CPL’s power demand level is satisfied accordingly. It is worth mentioning that hard switching without any filters or snubbers is used; therefore, noise and a small transient staring at each MOSFET switch is expected. The used semiconductors are also generic (regular Si and not SiC/GaN chemistry). Table 3 shows the efficiency at each level of power demand. The design of a high-efficiency production-level prototype is left for future research.

## 7. Conclusions

From the results provided in this paper, one can conclude that care must be taken during the design of controllers for the Boost converter, especially if the load can have several distant operating points or behave suddenly as a CPL or could occur conduction mode changes.

It was possible to corroborate both analytically and experimentally that the mathematical large-signal models presented here are accurate and could also be used to develop new predictive and robust strategies or for different control objectives. Regardless of the above, the nonlinear control law proposed here can stabilize the Boost converter with a CPL regardless of the conduction mode; this controller is easy and cheap to implement. Future research includes the analogous study of the stability for parallel boost stages, the analysis of robust stability, and the use of higher frequency with Sic or GaN devices to improve the converter’s efficiency.

## Figures and Tables

**Table 1:** Summary of the literature review.

References	Boost	Model type	CPL	Controller
[1,2,5]	✓	CCM		✓
[3,4]	✓	Static		
[6]	✓	CCM w/loss		
[7,8,9,11,12,13,18]	✓	Static		✓
[19]		CMI (implicit)		✓
[14]	✓	DCM w/loss		
[15]	✓	CRM		✓
[16]		DCM/CCM		
[17]	✓	DCM		✓
[20,22]	✓	DCM/CCM		
[21]	✓	DCM/CCM		✓
[23]	✓	Static		
[24]	✓	Static		✓
[25]		Static		✓
[26]	✓	DCM	✓	
[27]		CCM	✓	✓
[28]	✓	CCM	✓	✓
[29]		CCM	✓	✓
[30]		CCM	✓	✓
[33]	✓	CCM	✓	✓
[34]	✓	DCM	✓	✓
[35]	✓	CCM	✓	✓
This proposal	✓	CMI	✓	✓

**Table 2:** Current flow modes of the inductor.

${\bar{u}}_{1}$	${\bar{u}}_{2}$	Mode	Equations
*X*	0	Holding	$L\frac{d\hat{i}}{dt}=0$; $C\frac{d\hat{v}}{dt}=-\frac{P}{\hat{v}}$
0	1	Discharging	$L\frac{d\hat{i}}{dt}=-\hat{v}+E$; $C\frac{d\hat{v}}{dt}=\hat{i}-\frac{P}{\hat{v}}$
1	1	Charging	$L\frac{d\hat{i}}{dt}=E$; $C\frac{d\hat{v}}{dt}=\hat{i}-\frac{P}{\hat{v}}$

**Table 3:** Efficiency of the Boost converter operating in closed-loop.

CPL Power-Level (W)	Efficiency (%)	Conduction Mode
20	86.14	DCM
40	77.17	DCM
100	91.03	CCM
135	89.87	CCM

## Appendix A

### Appendix A.1. Proof of Proposition 1

**Proof.** From Theorem 4.7 in [40], if any eigenvalue of the Jacobian matrix has a positive real part in some operating point, then such an operating point is Lyapunov-unstable. Consider $u_{1}\to 0$ such that the equilibrium point is $i_{e}\to P/E$, $v_{e}\to E$. The Jacobian matrix at this operating point is:

(A1) $$J\approx \left[\begin{array}{cc} 0 & -\frac{1}{L}\\ \frac{1}{C} & \frac{P}{CE^{2}} \end{array}\right]$$

where d(sign(x))/dt=δ(x) is used, and δ is the Dirac’s delta function. The eigenvalues of *J* are:

(A2) $$\lambda_{1,2}\approx \frac{PL\pm \sqrt{L^{2}P^{2}-4LCE^{4})}}{2LCE^{2}}$$

of which, at least one has a positive real part with P,L>0, demonstrating instability of the operating point. □

### Appendix A.2. Proof of Theorem 1

**Proof.** From Theorem 8 in [38], consider the common Lyapunov candidate function $V=\frac{Cv^{2}}{L}+\frac{i^{2}}{2}$; hence, V(0)=0 and $V\in C^{\infty }$. For γ=1 and replacing Equation (25) on Equations (14) and (15), the time derivative along the system’s trajectory is sought to comply with:

$$\begin{array}{ccc} \dot{V} & = & -\frac{2CPv^{3}-C\Delta Eiv^{3}-\Delta Ek_{3}i^{2}v+Pk_{4}iv^{3}-\Delta Ek_{4}i^{2}v^{3}}{Lv(ik_{3}+Cv^{2}+ik_{4}v^{2}+Pk_{3}iv)} \end{array}$$

(A3) $$\begin{array}{ccc} & & -\frac{4LPfk_{3}i^{2}-CPk_{1}iv^{3}+CPk_{2}iv^{4}+4LPfk_{4}i^{2}v^{2}}{Lv(ik_{3}+Cv^{2}+ik_{4}v^{2})} \end{array}$$

(A4) $$\begin{array}{ccc} & & -\frac{4CLPfiv^{2}}{Lv(ik_{3}+Cv^{2}+ik_{4}v^{2})}<0 \end{array}$$

where $E\leq \Delta E=\left(1+2u_{1}-2u_{1}^{2}\right)E\leq \frac{3E}{2}$. Since v,i>0 the denominator is greater than zero, and using $k_{1},k_{4}$ from (26) and (27) one can look equivalently for:

(A5) $$\begin{array}{c} -\;CPk_{2}iv^{4}+\frac{9Ek_{3}}{16LPf}i^{2}v^{3}-2CPv^{3}-4CLPfiv^{2}-k_{3}Piv\\ -\;4LPfk_{3}i^{2}-\frac{3k_{3}}{2}i^{2}v^{2}+\frac{3k_{3}E}{2}i^{2}v<0. \end{array}$$

From the last two terms of the previous inequality, with $k_{3}>0$, and from expressions (26) and (27):

(A6) $$\begin{array}{ccc} -\frac{3k_{3}}{2}i^{2}v^{2}+\frac{3k_{3}E}{2}i^{2}v & < & 0\\ \frac{3k_{3}E}{2}i^{2}v & < & \frac{3k_{3}}{2}i^{2}v^{2}\\ Ei^{2}v & < & i^{2}v^{2}\\ E & < & v \end{array}$$

which is true for P>0; that is, in the Boost converter this condition is feasible under regular parameterization, and one looks for stability under this condition. Hence, inequality (A5) turns equivalently in:

(A7) $$\begin{array}{c} -CPk_{2}iv^{4}+\frac{9Ek_{3}}{16LPf}i^{2}v^{3}-2CPv^{3}-4CLPfiv^{2}-k_{3}Piv-4LPfk_{3}i^{2}<0. \end{array}$$

Taking the $v^{3}$ terms, one can find that their sum is negative if:

(A8) $$\begin{array}{ccc} \frac{9Ek_{3}}{16LPf}i^{2}v^{3}-2CPv^{3} & < & 0\\ i^{2} & < & \frac{32CLfP^{2}}{9Ek_{3}}. \end{array}$$

Note that $k_{3}>0$ can be arbitrarily small to allow any finite *i*, therefore inequality (A7) turns in:

(A9) $$\begin{array}{c} -CPk_{2}iv^{4}-4CLPfiv^{2}-k_{3}Piv-4LPfk_{3}i^{2}<0 \end{array}$$

which is always negative. On the other hand, for γ=0 and replacing Equation (25) on Equations (14) and (15), the time derivative along the system’s trajectory is sought to comply with:

(A10) $$\begin{array}{ccc} \dot{V} & = & Ei-2P+Pk_{1}i-Pk_{2}iv<0. \end{array}$$

Separating into two inequalities, one has:

(A11) $$\begin{array}{ccc} Ei-Pk_{2}iv & < & 0,\\ -2P+Pk_{1}i & < & 0. \end{array}$$

For the first one:

(A12) $$\begin{array}{c} Ei<Pk_{2}iE<Pk_{2}iv,\\ \frac{1}{P}<k_{2}, \end{array}$$

and for the second one:

(A13) $$\begin{array}{ccc} Pk_{1}i & < & 2P,\\ i & < & \frac{2}{k_{1}}. \end{array}$$

Since *V* is a common Lyapunov function, the proof is complete. □
